# Editorial: Ecology and Evolution of Coronaviruses: Implications for Human Health

**DOI:** 10.3389/fpubh.2022.926677

**Published:** 2022-05-20

**Authors:** Seth D. Judson, Vincent J. Munster

**Affiliations:** ^1^Department of Medicine, University of Washington, Seattle, WA, United States; ^2^Division of Intramural Research, Laboratory of Virology, National Institute of Allergy and Infectious Diseases, National Institutes of Health, Hamilton, MT, United States

**Keywords:** coronavirus, ecology, evolution, Middle East Respiratory Syndrome (MERS), SARS, COVID-19, spillover, SARS-CoV-2

Coronaviruses are diverse and ubiquitous viruses with the potential to cause significant morbidity and mortality among humans. The zoonotic potential of coronaviruses has been increasingly recognized with the global emergence of three diseases caused by coronaviruses in a span of 17 years–Severe Acute Respiratory Syndrome (SARS), Middle East Respiratory Syndrome (MERS), and coronavirus disease 2019 (COVID-19). Investigating the viruses that cause these diseases has greatly shaped our knowledge about the ecology and evolution of coronaviruses. The aim of this Research Topic is to explore how we can apply our growing knowledge about the ecology and evolution of coronaviruses to safeguard human health.

In November 2002, cases of pneumonia and respiratory distress were being reported in Guangdong province in southern China (1). By March 2003, a novel coronavirus, named SARS coronavirus (SARS-CoV), was isolated from patients with this disease (2). SARS-CoV was distantly related to known coronaviruses, including two human coronaviruses that cause mild respiratory illness (2). As the virus spread from southern China into Asia and North America, key questions arose. How did SARS-CoV emerge in human populations? What factors influenced virus transmission? And how did it cause acute respiratory distress?

Understanding the ecology and evolution of SARS-CoV became critical to answering these questions about emergence, transmission, and pathogenesis. Initial epidemiologic investigations supported the hypothesis that SARS-CoV was a zoonotic pathogen. Early human cases were more likely to have lived near live-animal markets and evidence of infection was found in Himalayan palm civets (*Paguma larvata*), racoon dogs (*Nyctereutes procyonoides*), and Chinese ferret-badgers (*Melogale moschata)* (1, 3). Years later, researchers found SARS-CoV-related viruses in *Rhinolophus* horseshoe bats in China, indicating that bats may be the reservoir hosts for SARS-CoV and that animals such as civets are intermediary hosts (4). At the beginning of the epidemic, SARS-CoV was found to be in high concentrations in the sputum of patients, suggesting that it was shed from the upper respiratory tract (2). The virus was also found to be stable in aerosol form and highly contagious (5), indicating a high likelihood of airborne transmission. On a molecular level, viral entry was found to depend on interactions between SARS-CoV's spike protein and the angiotensin-converting enzyme 2 (ACE2) receptor on human cells (6). Identifying potential reservoir hosts, modes of transmission, and molecular mechanisms of pathogenesis were essential to developing countermeasures for SARS.

A decade later, a patient in Saudi Arabia presented with pneumonia and respiratory distress, and a novel coronavirus, MERS-CoV, was isolated from his sputum (7). As cases spread beyond the Arabian Peninsula through travel and nosocomial transmission, understanding the emergence, transmission, and pathogenesis of MERS-CoV was essential to assessing its epidemic potential (8). In contrast to SARS-CoV, MERS-CoV was found to circulate in dromedary camels (9), predominantly replicate in the lower respiratory tract (10), and to rely on the DPP4 receptor (8, 11). These different aspects of the ecology and evolution of MERS-CoV have important implications for human health. Given frequent human contact with its reservoir host, MERS-CoV has a higher potential than SARS-CoV to spillover but it may be less transmissible, therefore influencing potential epidemic pathways (12).

When patients in Wuhan, China began developing pneumonia and respiratory distress in December 2019, a novel betacoronavirus was again identified as the etiologic agent (13). As SARS-CoV-2 spread across the globe, an unprecedented volume of research on coronaviruses was produced. Throughout the COVID-19 pandemic, the same questions regarding emergence, transmission, and pathogenesis have been at the core of this research. While SARS-CoV-2 initially appeared to have similar origins (14), aerosol stability (15), and receptors for cell entry as SARS-CoV (16), it was found to be more transmissible with increased spread from asymptomatic and presymptomatic individuals (17).

As shown by SARS, MERS, and COVID-19, understanding the ecology and evolution of coronaviruses is key to identifying factors that influence emergence, transmission, and pathogenesis–key determinants of infectious disease epidemics. The articles in this Research Topic further highlight this connection and provide insights into the future of coronavirus research. Jelinek et al. provide an in-depth review of the ecology and evolution of coronaviruses, presenting factors that influence spillover and zoonotic potential. This is complimented by work on identifying the emergence of novel coronaviruses. Kettenburg et al. identify novel betacoronaviruses in the subgenus *Nobecovirus* circulating in Malagasy bats, improving our phylogeographic knowledge of coronaviruses that may emerge in human populations. Nga et al. further examine the emergence of coronaviruses at the human-wildlife interface, identifying Sarbecoviruses in pangolins confiscated from the wildlife trade in Viet Nam. Factors influencing the transmission and pathogenesis of coronaviruses are revealed in Li et al.'s study on how variation in the cellular abundance of DPP4 influences MERS-CoV infection and pathogenesis.

Given what we have learned from the last two decades of coronavirus research, it is unlikely that SARS-CoV-2 is the last coronavirus to cause significant morbidity and mortality among humans. When novel coronaviruses emerge, it will be critical for rapid assessment of origins, transmission, and pathogenesis. Furthering our knowledge about the ecology and evolution of coronaviruses can help us answer these questions, but it will require interdisciplinary teams and studies that bridge the fields of ecology, evolutionary biology, virology, epidemiology, veterinary medicine, and human health.

## Author Contributions

All authors listed have made a substantial, direct, and intellectual contribution to the work and approved it for publication.

## Funding

VM is supported by the Division of Intramural Research at the NIAID.

## Conflict of Interest

The authors declare that the research was conducted in the absence of any commercial or financial relationships that could be construed as a potential conflict of interest.

## Publisher's Note

All claims expressed in this article are solely those of the authors and do not necessarily represent those of their affiliated organizations, or those of the publisher, the editors and the reviewers. Any product that may be evaluated in this article, or claim that may be made by its manufacturer, is not guaranteed or endorsed by the publisher.
